# Obituary

**Published:** 1861-10

**Authors:** 


### Obituary.

We have unhappily to record the loss of two well-known and highly honoured members of the profession—Professor QUEKETT, of the Royal College of Surgeons, and Dr. G. M. JONES, of Jersey.

A writer in the *Athenæum* states that Mr. Quekett began his career as a medical student at the London Hospital, and obtained by successful competition a studentship of anatomy, for three years, in the College of Surgeons; at the close of which, his superior attainments as an anatomist, especially in minute dissections and microscopical investigations, led to a permanent appointment in the Hunterian Museum. He was there principally occupied in extending and arranging the series of microscopical preparations; and the work on which his great reputation as a Histologist is chiefly based, is the "Illustrated Catalogue" of the specimens, showing the minute structure of tissues, in the College Museum, in Lincoln's-inn-fields. Mr. Quekett was appointed Professor of Histology; and on the retirement of Professor Owen in 1856, became principal Conservator of that museum. But his health rapidly failed, and after successive severe and debilitating attacks he expired at Pangbourne, on Tuesday, the 20th of August. Professor Quekett's published works on the microscope and microscopical anatomy have a high and deserved reputation; his great experience and vast extent of information made his opinion of peculiar value and in much request in obscure diseases and morbid alterations of structure; and the uniform readiness and urbanity with which he imparted his knowledge to all who visited, for that purpose, the museum of the Surgeons' College, will make the memory of this most worthy and valuable officer gratefully cherished. Physiological science and the medical profession have sustained a great loss in this excellent and, whilst health and strength were spared him, indefatigable man. Professor Quekett was selected by the council of the Royal Society from the candidates for fellowship, and was elected in 1860.

The following account of Mr. Jones is from the *Jersey Independent* (Sept. 13th):—

George Matthew Jones was the second son of the late Charles Jones, M.D., of this place. He was born on the 14th of March, 1805, and in early youth commenced the study of his profession. Our local records tell us that at the age of twenty-two he was gazetted as Assistant-Surgeon to one of the Militia Regiments of this island. Commencing the pursuit of the profession he was destined to adorn, under the happiest auspices of his esteemed and respected, as well as distinguished father; and being possessed of an acute and discerning intellect, as well as an untiring zeal and energy, his success was assured. We find him at the age of twenty obtaining distinguished honours at the University of Edinburgh. He entered this medical school the year before, and during the whole period of his pupilage resided in the house of the celebrated Dr. James Hamilton, with whom he was an especial favourite. His aptitude for work soon gained him many friends, and on the completion of his second year's study, we find him taking the first honours of the school for his anatomical dissections and dissertations. In 1826 he took his diploma of Surgeon, and returned to his native place, where he soon became known as a talented and promising practitioner, succeeding his amiable and accomplished father, whose extensive connexion opened a brilliant prospect for the son.

In 1833 he was appointed Surgeon to the Ordnance, and some years later to the General Hospital, and afterwards to the Prison and House of Correction. It is needless to trace from year to year the progress of his professional career. Shortly after his appointment to the Hospital, he commenced a series of medical and surgical reports, in the preparation of which his intellect found fitting occupation, and which no doubt laid the foundation of that clear, lucid, and graphic style of writing which ever afterwards characterized his professional dissertations and contributions to medical literature.

In the year 1843 he contributed to the Medical Journals some interesting cases, the features and details of which at once stamped him as an acute observer, a bold and successful surgeon, as well as a sound practitioner. In the year 1853 he commenced those operations for the cure and recovery of diseased joints and preservation of the limb which, until their revival in 1850 by the distinguished Professor Ferguson, of King's College, had been deemed, in consequence of the comparative failures of Park and Moreau—"the opprobria of Surgery." Without discussing the merits of this order of treatment which its foremost and most sanguine partisans still admit to be *sub judice,* it is certain that the operations performed by the subject of this notice have gone far towards solving the problem; they certainly prove the feasibility of the method, and must be regarded as brilliant achievements in Surgery. Those achievements gained Mr. Jones an European reputation. His fame was reflected on the Jersey Hospital, which acquired a celebrity possessed by but few provincial institutions of the like character—a reputation, we must say, well-deserved, considering the liberal and generous treatment experienced within its walls by suffering humanity.

In 1855 the Royal College of Surgeons of England summoned Mr. Jones to London, and conferred upon him their diploma, intending, had he lived, to follow up this distinction by the still greater one of their Fellowship; the rules of that body rendering a certain interval imperative, else the highest honour would at once have been conferred. Other honours followed: to wit, the Honorary Fellowship of the Medical Society of London, that of the Medical Society of Paris, and of the Surgical Association of Berlin.

On the death of Dr. Macreight, Mr. Jones was appointed Medical Inspector-General of Militia, and by his extensive and daily increasing practice, continued to receive additional proofs of the high consideration and confidence reposed in his distinguished surgical skill. About this time he performed several difficult and dangerous surgical operations in which he was very fortunate; and as one of his sayings was "nothing venture nothing have," the boldness with which he encountered difficulty almost gave the stamp of originality to his achievements. It is only asserting a truism that his eminent professional abilities were acknowledged, not only by the profession, but by the public at large; so that subscriptions were soon raised, both in the Island, in England, France, and elsewhere, for a marble bust originally intended to be placed in the board-room of the General Hospital, but in consequence of the destruction by fire of that institution, it was provisionally deposited at his own house, to be placed at its intended location as soon as the Hospital is restored. This beautiful work of art comes from the studio of Patrick Macdowell, R.A. It is an excellent likeness, full of character and energy, and adds to the renown of the already celebrated sculptor.

A long and painful illness incapacitated Mr. Jones for a time for the active duties of his profession, but detracted nothing from his well-earned fame. He was always highly esteemed by his professional brethren for his sagacious as well as quick discernment of disease, and he was in the height of his happiness when surrounded by a numerous clinique, and exhibiting his cases, he received a stimulus to further exertion from their approval and applause.

